# Stage-Informed *Trypanosoma cruzi* Antigen Selection Enhances Assay Concordance in the Evaluation of Discordant Chagas Disease Serology

**DOI:** 10.3390/microorganisms14091899

**Published:** 2026-08-27

**Authors:** Luis Adrián De Jesús-González, Ignacio Martínez, Jorge Procopio-Velázquez, Muslim Schabib-Hany, Bertha Espinoza

**Affiliations:** 1 Unidad de Investigación Biomédica de Zacatecas, Instituto Mexicano del Seguro Social, Zacatecas 98000, Mexico; 2 Departamento de Inmunología, Instituto de Investigaciones Biomédicas, Universidad Nacional Autonóma de Mexico (UNAM), Mexico City 04510, Mexico; imm@iibiomedicas.unam.mx; 3 Hospital de Infectología, Centro Médico Nacional “La Raza”, Instituto Mexicano del Seguro Social, Mexico City 02990, Mexico; jorge.procopio@imss.gob.mx (J.P.-V.);

**Keywords:** Chagas disease, *Trypanosoma cruzi*, serological diagnosis, indeterminate serology, trypomastigote antigen, ELISA, Western blot

## Abstract

Chagas disease is difficult to diagnose in the chronic phase, especially when serological assays disagree. We examined whether *Trypanosoma cruzi* metacyclic trypomastigotes’ antigenic extracts may increase ELISA/Western blot concordance in initially discordant serum compared to epimastigotes. A total of 178 human blood samples were studied, including seropositive persons, Chagasic cardiomyopathy patients, discordant sera, seronegative controls, and sera from various parasitic illnesses. Antigenic extracts from epimastigotes and metacyclic trypomastigotes were extracted using sonication or urea, and Western blot and ELISA were used to compare their behavior. Sonicated metacyclic trypomastigote extracts produced concordant ELISA/Western blot results in 31/35 originally discordant sera, compared to 19/35 for sonicated epimastigotes, 20/35 for urea-extracted trypomastigotes, and 17/35 for urea-extracted epimastigotes. All extracts in the serum panel previously classified using the standardized in-house serological platform showed 100% positive percent agreement, but negative percent agreement varied between 93.3% for sonicated metacyclic trypomastigotes and 66.7% for urea-extracted epimastigotes. These estimates reflect agreement with the previous serological classification, not diagnostic accuracy versus an independent reference standard. Cross-reactivity was mainly observed in sera from patients with leishmaniasis, while sera from the cardiopathic group showed an exploratory preferential recognition pattern with bands of 18, 22, 24, and 26 kDa in the sonicated metacyclic trypomastigote preparation. The comparison did not account for potential clinical variables or molecularly identify these bands. These data demonstrate that stage-informed antigen selection, particularly using sonicated metacyclic trypo-mastigote extracts, can affect serum panel assay concordance and nonspecific reactivity.

## 1. Introduction

Chagas disease, or American trypanosomiasis, is a primary neglected tropical disease caused by the hemoflagellate parasite *Trypanosoma cruzi*. It was first described in 1909 by Brazilian physician Carlos Chagas, who identified the parasite, its transmission cycle, and its pathological impact on humans [1,2]. Today, this disease remains a public health problem in Latin America, with approximately 8 million people estimated to be infected with *T. cruzi* worldwide, mainly in endemic areas of continental Latin America [3]. However, due to migration, Chagas disease has expanded to non-endemic areas, including Europe, Australia, Japan, and Canada, underscoring the need for improved diagnostic and control strategies [4].

Transmission of *T. cruzi* occurs mainly through blood-sucking insect vectors of the subfamily Triatominae (family Reduviidae) [5]. However, there are other transmission routes, such as blood transfusion, organ transplantation, congenital transmission, ingestion of contaminated food, and accidental exposure in laboratories [6]. The disease progresses in two phases: an acute phase, which may be asymptomatic or present mild symptoms such as fever and inflammation at the inoculation site, and a chronic phase, which may persist for decades. Approximately 30 to 40% of infected individuals develop serious complications, including Chagasic heart disease, digestive disorders (megaesophagus, megacolon), and even sudden death due to cardiac arrhythmias [2,3].

Diagnosis of Chagas disease is essential for adequate clinical management, preventing progression, and reducing the risk of transmission [7]. The choice of diagnostic methodology depends on the disease’s stage. In the acute phase, high parasitemia allows direct parasitological methods, such as microscopic observation, blood culture, and polymerase chain reaction (PCR) [8]. However, in the chronic phase, parasitemia is low and intermittent, making direct detection of the parasite difficult and serological tests the diagnostic standard [9]. The World Health Organization recommends using at least two serological tests, such as ELISA, Western blot, indirect immunofluorescence, and hemagglutination [10]. However, one of the main challenges in serological diagnosis is the presence of doubtful or discordant cases, in which test results are inconsistent. This diagnostic uncertainty complicates clinical decision-making, especially in detecting infected blood donors, monitoring congenital transmission, and selecting patients for treatment [11,12].

One factor contributing to discrepancies among serological tests is antigen selection. Most commercial kits use antigens derived from epimastigotes, the replicative form present in the insect vector, because these parasites can be readily cultured in vitro [13]. However, epimastigotes are not present in the mammalian host. By contrast, trypomastigote stages are directly encountered by the host during infection: metacyclic trypomastigotes initiate mammalian infection, whereas bloodstream trypomastigotes circulate during the acute phase. Consequently, sera from infected individuals may recognize a broader or different repertoire of host-exposed antigens that are absent or underrepresented in conventional epimastigote preparations. Accordingly, trypomastigote-derived antigens have been proposed to improve the sensitivity and specificity of serological assays [14]. Several studies have also suggested that these preparations may reduce cross-reactivity with other parasites, particularly *Leishmania* spp., which share antigenic determinants with *T. cruzi* [15,16,17]. In addition, Western blot analyses have shown that sera from patients with different clinical manifestations of Chagas disease recognize distinct antigenic profiles depending on the parasite stage used [15,16]. Antigen representation may be further influenced by the extraction procedure because different physicochemical methods recover soluble, hydrophobic, membrane-associated, or otherwise poorly solubilized proteins with different efficiencies. Therefore, both the parasite stage and the extraction method can modify the abundance and accessibility of diagnostically informative or cross-reactive epitopes, causing the same serum sample to show different reactivity across antigenic preparations [18].

In this study, we evaluated antigenic extracts from epimastigotes and trypomastigotes of *T. cruzi* and compared protein patterns obtained by sonication and urea extraction. In addition, we analyzed their diagnostic performance using Western blot and ELISA to determine their usefulness in resolving cases of doubtful serology. Our results suggest that extracts from trypomastigotes, particularly those obtained by sonication, may be a promising tool to improve the accuracy of Chagas disease diagnosis in samples with indeterminate serological results. This study emphasizes the importance of selecting appropriate antigenic targets to optimize the reliability of serological tests and contribute to developing more effective diagnostic strategies for controlling Chagas disease.

## 2. Materials and Methods

### 2.1. Serum Sample Collection

A total of 156 archived serum samples were obtained through collaborating clinical institutions in central Mexico, including the Blood Bank of Centro Médico Nacional “La Raza” of the Instituto Mexicano del Seguro Social and the Instituto Nacional de Cardiología Ignacio Chávez. The samples were subsequently transferred to, characterized, and maintained in the serum bank of Dr. Bertha Espinoza at the Instituto de Investigaciones Biomédicas, Universidad Nacional Autónoma de México. Thus, the Instituto de Investigaciones Biomédicas served as the serum repository and reference laboratory rather than as the site of patient recruitment. Of these samples, 68 corresponded to individuals with positive serology for *T. cruzi* in an indeterminate stage, 38 to patients with Chagasic cardiomyopathy, 35 to cases with discordant serology, and 15 to individuals with negative serology. In addition, 22 samples from patients with other parasitic infections (toxoplasmosis, n = 3; taeniasis/cysticercosis, n = 4; and leishmaniasis, n = 15) were included to evaluate cross-reactivity. Although the serum panel originated from more than one clinical institution, both source institutions are located in central Mexico; therefore, the cohort should be considered regionally derived rather than geographically representative.

Serological categorization was performed using the in-house diagnostic platform developed by our group to detect anti-*T. cruzi* antibodies, employing Mexican parasite strains as antigenic sources [19]. This platform was previously standardized using 246 serum samples and was evaluated through a double-blind interlaboratory comparison of 50 sera against previously established serological assays performed at the Instituto Nacional de Cardiología Ignacio Chávez, showing high concordance between laboratories (κ = 0.96) [19]. Therefore, the initial serum categories used in the present study were not generated *de novo* but were derived from a previously standardized serological framework. Nevertheless, because no additional independent clinical, commercial, molecular, or parasitological reference standard was applied contemporaneously to the present serum panel, subsequent analyses were interpreted as comparative, reference-relative assessments of antigenic preparations.

Previously classified seropositive sera were defined as samples with concordant ELISA and Western blot reactivity within this standardized serological framework, whereas previously classified seronegative controls were defined as samples lacking reactivity in both assays. These categories were used as the comparative serological reference for the present study and were not considered equivalent to classifications established through an independent contemporaneous reference standard.

Discordant sera were defined as samples with non-concordant ELISA and Western blot results in this previously validated in-house diagnostic procedure, encompassing ELISA-negative/WB-positive and ELISA-positive/WB-negative patterns. Crucially, these samples were not presupposed to represent true-positive or true-negative cases; rather, they were examined as unresolved serological samples to assess whether alternative antigenic preparations from various parasite stages could increase concordance between ELISA and Western blot after retesting. Concordance obtained after retesting was not interpreted as confirmation of the true infection status of the corresponding individuals.

Demographic and clinical data, including age, sex, clinical presentation, electrocardiographic abnormalities, and prior etiological treatment history, when applicable, were compiled for each study cohort. The absence of comprehensive clinical documentation for stored sera was taken into account when interpreting the results.

Information on concomitant illnesses other than the diagnoses used to define the study groups was not consistently available for the archived sera. Therefore, the potential influence of comorbidities on antibody reactivity could not be systematically evaluated. The sera from patients with toxoplasmosis, taeniasis/cysticercosis, and leishmaniasis constituted a separate exploratory cross-reactivity panel and did not represent documented concomitant infections in the primary study groups.

### 2.2. Culture of Trypanosoma cruzi Epimastigotes

Epimastigotes of *T. cruzi* strain Querétaro (TBAR/MX/0000/Querétaro) were cultured in LIT (Liver Infusion Tryptose) medium supplemented with 10% inactivated fetal bovine serum (FBS) at 56 °C for 30 min and 25 µg/mL hemin. Parasites were plated at a density of 1 × 10^6^ parasites/mL and cultured for 4 days until reaching the exponential phase, with a final density of 40 × 10^6^ parasites/mL.

The Querétaro strain, classified as *T. cruzi* discrete typing unit I (TcI), was selected because it is a Mexican strain previously used to standardize ELISA and Western blot assays for Chagas disease serodiagnosis in Mexico [19]. Both epimastigote and metacyclic trypomastigote preparations were generated from the same Querétaro TcI strain. Its use in the present study ensured continuity with a locally standardized serological platform while extending the antigen-source comparison from conventional epimastigote-derived extracts to metacyclic trypomastigote-derived preparations. Both epimastigote and metacyclic trypomastigote preparations were generated from the same Querétaro strain. Therefore, parasite genetic background was held constant in the within-study comparisons between life-cycle stages. This design permits the identification of stage-associated differences within the Querétaro TcI background but does not establish that the same effects occur in other strains or DTUs.

### 2.3. Induction of Metacyclogenesis

Stationary-phase epimastigotes were induced to differentiate into metacyclic trypomastigotes by nutritional stress and transfer to serum-free Grace medium. Briefly, parasites were harvested from stationary-phase LIT cultures, washed twice with PBS by centrifugation at 1000× *g* for 10 min, and resuspended in Grace medium without FBS at a final concentration of 250 × 10^6^ parasites/mL. Cultures were incubated under these conditions for up to 12 days, and metacyclogenesis was monitored at the indicated time points. The percentage of metacyclic trypomastigotes was estimated using a complement-mediated lysis assay with fresh guinea pig serum, as previously described by Arroyo-Olarte et al. [20], based on the relative resistance of metacyclic forms to complement-dependent lysis. Metacyclogenesis efficiency was determined from six independent experiments, with each condition evaluated in triplicate.

Note: Day 0 corresponded to the stationary-phase epimastigote inoculum immediately after transfer to serum-free Grace medium and was considered the experimental baseline rather than a post-induction time point. Day 4 was the first predefined post-induction sampling point, followed by days 7 and 12 to evaluate the progression and subsequent stabilization of metacyclogenesis. Earlier post-induction time points were not included in the experimental sampling schedule.

### 2.4. Obtaining Antigenic Extracts

Protein extracts were prepared from epimastigotes and metacyclic trypomastigotes using sonication and urea extraction. Four antigenic preparations were evaluated: sonicated metacyclic trypomastigotes (TS), sonicated epimastigotes (ES), urea-extracted metacyclic trypomastigotes (TU), and urea-extracted epimastigotes (EU). These abbreviations are used throughout the manuscript.

#### 2.4.1. Sonication

The parasites were washed with PBS and resuspended in 10 mM Tris-HCl buffer (pH 8.2) with protease inhibitors (12 µM EDTA, 1 mM PMSF, 100 µM leupeptin, and 1 µM pepstatin). They were subjected to sonication (Vibra-Cell™ ultrasonic processor (Sonics and Materials, Inc., Newtown, CT, USA)) at 100 watts for 4 min, in 4 cycles of 1.5 s each. The mixture was centrifuged at 10,000× *g* for 30 min at 4 °C, and the supernatant was recovered.

#### 2.4.2. Urea Extraction

Parasites were washed with PBS and resuspended in a lysis solution containing 7 M urea, 2 M thiourea, 4% CHAPS (3-[(3-cholamidopropyl)dimethylammonio]-1-propanesulfonate, a zwitterionic detergent), and the protease inhibitors listed above. They were incubated on ice with shaking and centrifuged at 12,800× *g* for 15 min at 4 °C.

The protein concentration in each extract was determined using the DC Protein Assay (Bio-Rad Laboratories, Hercules, CA, USA) for sonicated extracts and the 2-D Quant Kit (Amersham Biosciences, Uppsala, Sweden) for urea-derived extracts.

### 2.5. SDS-PAGE

Protein extracts were analyzed on 12% polyacrylamide gels under denaturing conditions (SDS-PAGE) using the Mini-PROTEAN^®^ Tetra Cell system (Bio-Rad Laboratories, Hercules, CA, USA). A total of 10 µg of protein was loaded per lane, and electrophoresis was performed at 200 V for 1 h. SDS-PAGE gels were digitized, and detectable protein bands were analyzed according to their apparent molecular weight using myImageAnalysis™ software, version 2.0 (Thermo Fisher Scientific, Waltham, MA, USA). For comparative purposes, each band was scored qualitatively as present or absent in each antigenic preparation. Detectable bands were assigned a value of 1, whereas non-detectable bands were assigned a value of 0. The resulting binary dataset was used to calculate the total number of detectable bands per extract and to generate a presence/absence matrix using GraphPad Prism version 9.0 (GraphPad Software, San Diego, CA, USA). This analysis was intended to compare qualitative differences in band distribution and apparent protein complexity among antigenic preparations.

### 2.6. Immunoblotting

Proteins separated by SDS-PAGE were transferred onto nitrocellulose membranes (Schleicher and Schuell, Dassel, Germany) at 100 V for 1 h using a Mini Trans-Blot system (Bio-Rad Laboratories, Hercules, CA, USA). Membranes were blocked overnight at 4 °C in 10% skimmed milk in PBS, then incubated individually with test sera diluted 1:500 in blocking solution for 2 h at 37 °C. After washing with PBS containing 0.05% Tween 20, membranes were incubated with peroxidase-conjugated anti-human IgG (1:10,000) for 2 h at room temperature. Immunoreactive bands were visualized using 3,3′-diaminobenzidine and hydrogen peroxide.

For diagnostic immunoblotting, an antigen load of 3.5 µg per strip has been routinely used in our laboratory under previously standardized conditions for *T. cruzi* serology [19]. In the present study, however, immunoblot assays were also performed using 10 µg of antigen to increase antigen availability in sera with discordant serology, with the aim of improving the resolution of unresolved cases and determining whether the specificity of metacyclic trypomastigote-derived extracts was preserved under higher antigen-loading conditions.

The reactivity of the Western blot was assessed using parameters previously established for Mexican *T. cruzi* antigenic extracts [20]. Immunoreactive bands identified in negative-control samples were deemed nonspecific and omitted from the final diagnostic assessment. A sample was deemed WB-positive if at least one *T. cruzi*-specific immunoreactive band was identified, following the removal of nonspecific bands. The band assignment was determined by comparison with molecular weight markers and the positive and negative control sera included in each experiment.

In the comparative analysis, identified bands were evaluated by apparent molecular weight, and the resulting banding patterns were used to compare antigenic preparations, discordant sera, cross-reactivity panels, and clinical groups. Representative Western blot images are shown in the main figures. The available original, uncropped immunoblot scans recovered from the archived dataset are provided in the Supplementary Materials. During figure preparation, images were cropped only for presentation. No selective or nonlinear image modifications were performed, and any brightness or contrast adjustments were linear and applied uniformly to the entire corresponding blot panel.

For the comparison of cardiopathic and non-cardiopathic sera presented in Section 3.4, the strips corresponding to the four antigenic preparations (TS, ES, TU, and EU) tested with each serum were processed in parallel under identical incubation and detection conditions. All sera included in this comparison had previously been classified as T. cruzi-seropositive using the standardized conventional serological framework, based on concordant ELISA and Western blot reactivity [19]. Therefore, the immunoblots performed in the present study constituted a repeat evaluation of previously characterized sera using the four antigenic preparations. This prior testing confirmed seroreactivity; however, because it was performed earlier and did not use the same gels or blots as the current four-preparation comparison, it was not considered a technical replicate to control inter-gel variability. Because of the number of sera evaluated, the complete set of cardiopathic and non-cardiopathic sera could not be analyzed on a single gel or blot or within the same experimental run; instead, sera from the two clinical groups were evaluated using strips obtained from separate gels and blots. Systematic technical replication of the present four-preparation immunoblot analysis was not performed. Molecular-weight markers and positive and negative control sera were included in each experiment for band assignment. Consequently, comparisons between the clinical groups were based on qualitative band presence or absence and recognition frequencies, and the representative blots were not used for densitometric comparisons of band intensity across gels.

### 2.7. Enzyme-Linked Immunosorbent Assay

ELISA plates (Costar 3590, Corning Incorporated, Corning, NY, USA) were coated with 100 µL of antigenic extract at 10 µg/mL in carbonate-bicarbonate buffer (pH 9.6). Plates were then blocked with 1% bovine serum albumin in PBS for 2 h at 37 °C and subsequently incubated overnight at 4 °C with test sera diluted 1:500 in PBS containing 0.05% Tween 20. After washing, peroxidase-conjugated anti-human IgG diluted 1:7500 was added and incubated for 2 h at 37 °C. The enzymatic reaction was developed with *o*-phenylenediamine and stopped with 2.5 N H_2_SO_4_. Absorbance was measured at 490 nm using a Bio-Rad Model 550 microplate reader. The cutoff value was defined as the mean optical density of negative control sera plus 2.5 standard deviations [19].

This cutoff criterion was retained from the previously standardized in-house ELISA procedure using Mexican *T. cruzi* antigenic extracts [19]. The mean optical density of negative-control sera plus 2.5 standard deviations was set as a conservative threshold to minimize false-positive reactions while maintaining the ability to identify chronic symptomatic and asymptomatic *T. cruzi* infection. ELISA results were reported as the OD490-to-cutoff ratio to enable comparison among antigenic preparations.

### 2.8. Statistical Analyses

All statistical analyses were performed using GraphPad Prism version 9.0. Data are presented as mean ± standard deviation (SD) unless otherwise indicated. Differences in metacyclogenesis percentages across time points were evaluated using an ordinary one-way analysis of variance (ANOVA), followed by Tukey’s multiple comparisons test. ELISA reactivity among initially discordant sera, expressed as the OD490-to-cutoff ratio, was compared across the four antigenic preparations using the Friedman test because the same 35 sera were evaluated under all four conditions and the data did not satisfy the assumptions required for parametric repeated-measures analysis. Specifically, Shapiro–Wilk testing indicated departures from normality for the ES and EU preparations, and dispersion differed among conditions. Dunn’s multiple-comparisons test was used for post hoc pairwise comparisons. Positive and negative control sera were displayed as contextual reference groups in the corresponding figure but were not included in the inferential comparison among TS, ES, TU, and EU.

Because the number of immunoreactive bands constitutes discrete count data, comparisons of band counts were analyzed using non-parametric tests. Comparisons between two clinical groups, such as cardiopathic versus non-cardiopathic seropositive individuals, were performed using the Mann–Whitney U test. Comparisons among multiple antigenic preparations were performed using the Kruskal–Wallis test, followed by Dunn’s multiple-comparisons test. Graphical representations included summary distributions and individual-value plots to illustrate the dispersion of data across groups.

Comparisons of the number of antigenic bands recognized according to clinical status (cardiopathic vs. non-cardiopathic) and antigenic extract (TS, ES, TU, and EU) were analyzed using two-way ANOVA, followed by Šídák’s multiple comparisons test for pairwise comparisons within each clinical group. Graphical representations included both summary distributions and individual-value plots to illustrate the dispersion of data across groups.

Comparative agreement with the previous classification established by the standardized in-house serological platform was evaluated using 2 × 2 contingency analyses based exclusively on Western blot results obtained with 10 µg of antigen. Positive percent agreement (PPA) was calculated as the proportion of previously classified seropositive sera that were reactive with each antigenic preparation, whereas negative percent agreement (NPA) was calculated as the proportion of previously classified seronegative sera that remained non-reactive. Exact 95% confidence intervals for PPA and NPA were calculated using binomial methods. Because no additional independent clinical, commercial, molecular, or parasitological reference standard was available for the present serum panel, these estimates were interpreted as reference-relative agreement measures rather than as definitive estimates of diagnostic sensitivity or specificity. Positive and negative predictive values were not calculated. ELISA was not included in the contingency-based agreement analysis; instead, it was used together with Western blot to assess changes in assay concordance among initially discordant sera. A *p*-value < 0.05 was considered statistically significant.

## 3. Results

### 3.1. Induction of Metacyclogenesis In Vitro

Obtaining metacyclic trypomastigotes under laboratory conditions is challenging, as this form of the parasite is found in the hemolymph of the *Triatominae* vector, making mass production difficult [21]. Due to this limitation, most diagnostic studies use epimastigotes, as this phase is less complicated to culture in axenic media. However, epimastigotes do not represent the infective form of the parasite in the mammalian host, which may compromise the sensitivity and specificity of serological assays [19,22].

In this study, we successfully induced metacyclogenesis of *T. cruzi* from epimastigotes using a homemade protocol based on nutritional stress and the simulation of vector hemolymph conditions. To do this, epimastigotes were cultured in LIT medium until reaching the stationary phase, then transferred to Grace medium, which has been shown to favor epimastigote differentiation into trypomastigotes by mimicking the composition of insect hemolymph [23].

**Figure 1:**
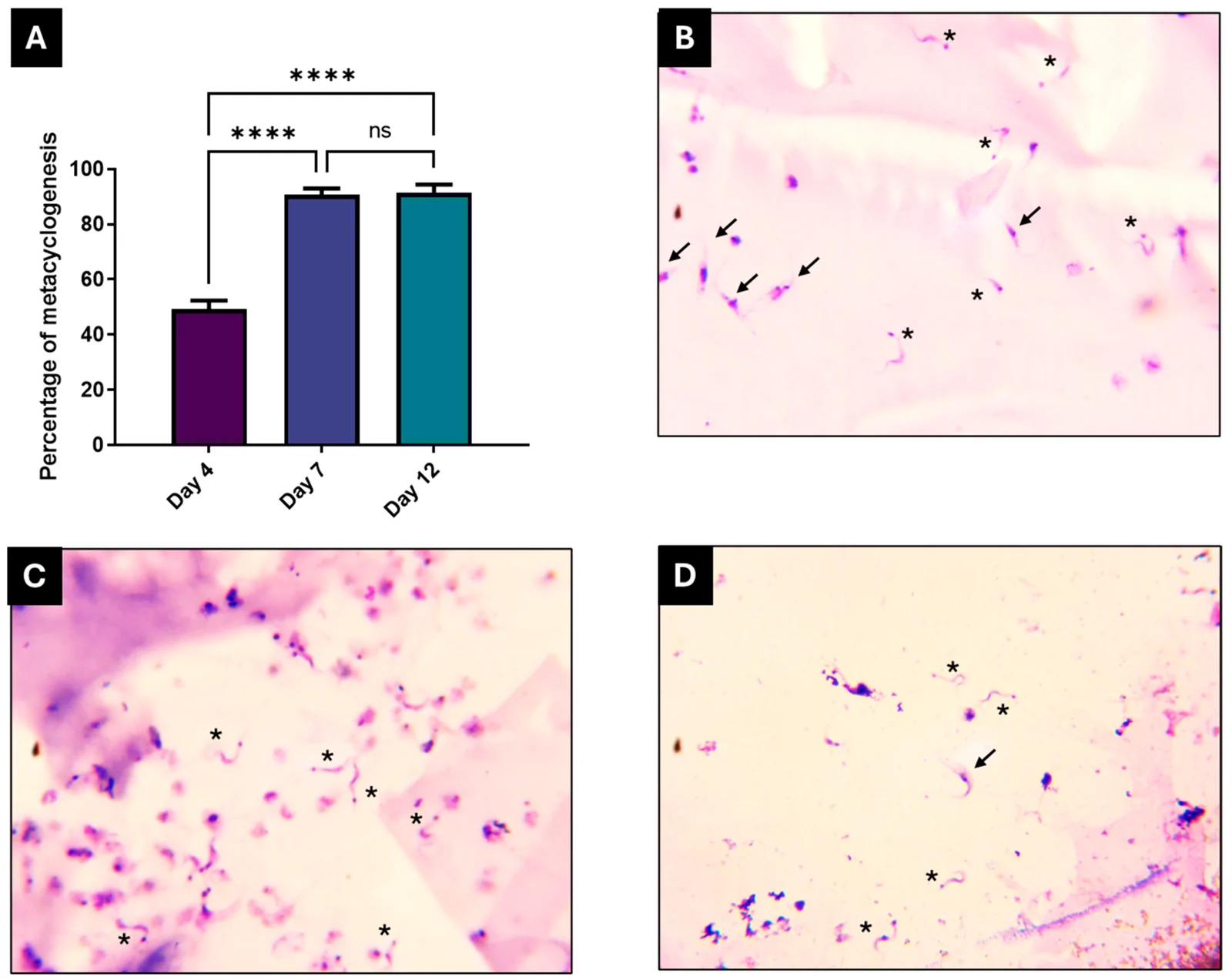
**Progressive in vitro metacyclogenesis of** ***Trypanosoma cruzi*** **under nutritional stress.** (**A**) Percentage of metacyclogenesis at days 4, 7, and 12, determined by complement lysis assay. ****, *p* < 0.0001, ns: not significant, (**B**–**D**) Representative Giemsa-Wright-stained parasites at day 4 (**B**), day 7 (**C**), and day 12 (**D**), illustrating the progressive transition from epimastigote to trypomastigote forms during metacyclogenesis. Arrows indicate epimastigotes, in which the kinetoplast is located between the nucleus of the parasite and the flagellum, whereas asterisks indicate trypomastigotes, in which the kinetoplast is located between the nucleus and the posterior end of the parasite.

The highest conversion rate to metacyclic trypomastigotes (91.55% ± 0.38) was obtained by culturing epimastigotes in Grace medium without FBS at a density of 250 × 10^6^ parasites/mL for 7 days (Figure 1). This efficiency was calculated from six independent experiments, each performed in triplicate. To confirm the transformation of epimastigotes to trypomastigotes, a complement lysis assay was performed using fresh guinea pig serum. It was observed that parasites differentiated under optimal conditions showed high resistance to lysis, a characteristic of the metacyclic form of *T. cruzi* (Figure 1B–D) [24,25].

Together, these data confirm that the Grace medium/nutritional-stress protocol generated reproducible metacyclogenesis and provided sufficient metacyclic trypomastigote material for antigenic extract preparation.

### 3.2. Epimastigote- and Trypomastigote-Derived Extracts Displayed Distinct Protein Profiles

To compare the antigenic composition of the different parasite extracts, SDS-PAGE was performed using 10 µg of total protein per lane, a higher antigen load than that routinely used for diagnostic immunoblotting in our laboratory (3.5 µg of antigen), in order to maximize band detection and to assess whether increased antigen availability modified protein complexity while preserving the diagnostic specificity of trypomastigote-derived preparations. Sonication was included because it represents the conventional extraction approach previously used in our laboratory for *T. cruzi* serodiagnostic antigen preparation [19], whereas the urea/thiourea/CHAPS-based protocol was additionally evaluated to determine whether a chaotropic extraction strategy could recover protein species that are less efficiently solubilized under standard aqueous lysis conditions, including proteins with greater hydrophobic character or stronger membrane association [26].

**Figure 2:**
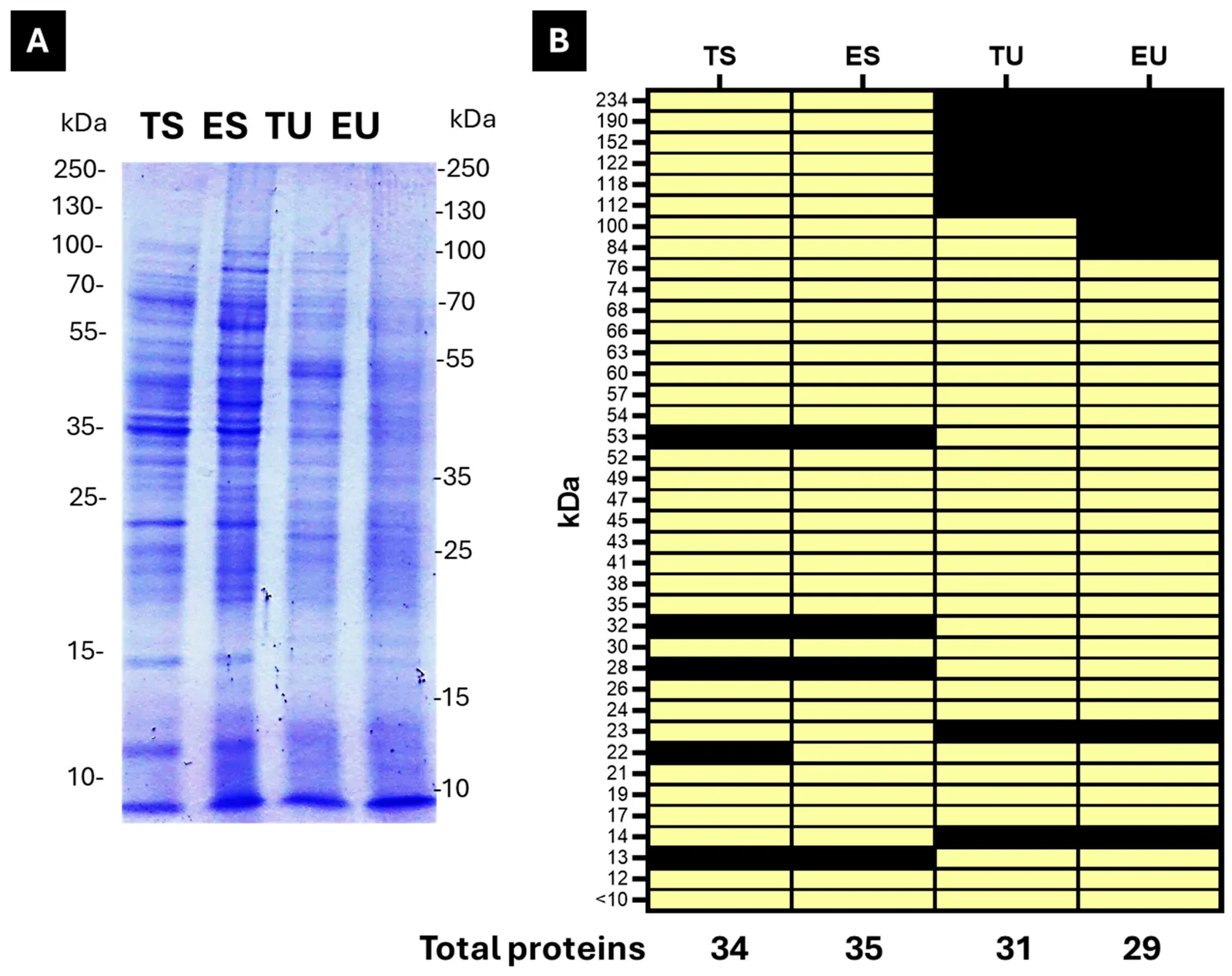
**Comparative protein banding patterns of** ***T. cruzi*** **antigenic extracts.** (**A**) SDS-PAGE of sonicated and urea-extracted preparations obtained from metacyclic trypomastigotes and epimastigotes, using 10 µg of total protein per lane. (**B**) Binary presence/absence matrix summarizing detectable protein bands across the four antigenic preparations according to apparent molecular weight. Yellow denotes the presence of a detectable band, and black denotes its absence. This matrix was used to compare the qualitative band distribution and the total number of bands. TS, sonicated metacyclic trypomastigotes; ES, sonicated epimastigotes; TU, urea-extracted metacyclic trypomastigotes; EU, urea-extracted epimastigotes.

Protein patterns differed according to both parasite stage and extraction method (Figure 2A). Sonicated epimastigote extracts displayed the highest qualitative protein complexity, with 35 detectable bands, followed by sonicated metacyclic trypomastigote extracts, with 34 bands. Urea-extracted trypomastigotes and epimastigotes yielded 31 and 29 detectable bands, respectively. Although the urea-based protocol revealed a limited subset of bands not observed in sonicated preparations, the total number of detectable bands was lower than that observed with sonication. To summarize these qualitative differences, a binary presence/absence matrix was generated according to apparent molecular weight (Figure 2B). This analysis showed that the four antigenic preparations differed not only in the total number of bands but also in the distribution of detectable bands across molecular-weight regions. Together, these findings indicate that, within the same Querétaro TcI genetic background, both parasite stage and extraction procedure influenced the qualitative composition of the antigenic preparations used for comparative serological evaluation.

### 3.3. Sonicated Metacyclic Trypomastigote Extracts Increased Assay Concordance Among Initially Discordant Sera

The comparative behavior of the four antigenic preparations was evaluated in 35 sera with discordant serology, defined as unresolved samples that yielded non-concordant ELISA and Western blot results within the previously standardized in-house diagnostic platform used in our laboratory for chronic *T. cruzi* serology [19]. Among these 35 initially discordant sera, 27 had previously shown an ELISA-negative/Western blot-positive pattern, whereas the remaining eight had shown an ELISA-positive/Western blot-negative pattern (Table 1). This distinction is clinically relevant because confirmed chronic Chagas disease generally requires reactivity in two different serological assays, whereas discrepant samples remain unresolved unless an additional confirmatory strategy is applied [27,28,29,30,31]. These samples were not considered true positives or true negatives a priori; rather, they were analyzed as unresolved serological cases to determine whether alternative antigenic preparations could increase ELISA/Western blot concordance after retesting. Such concordance was interpreted as improved agreement between assays and not as determination of the true infection status of the individuals.

**Figure 3:**
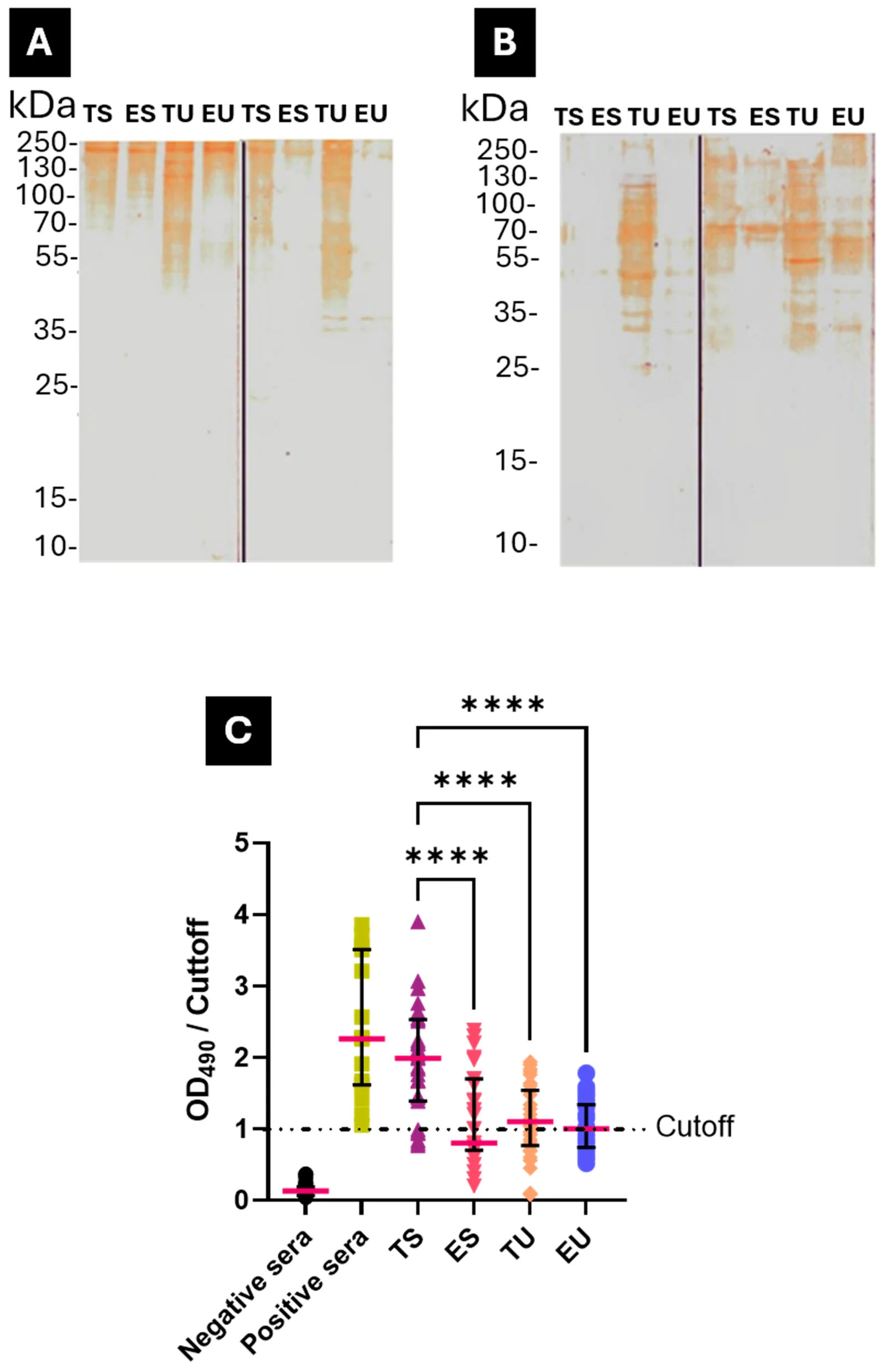
**Representative immunoblot and ELISA analysis of sera with discordant Chagas serology.** (**A**) Western blot profiles of two ELISA-negative/Western blot-positive sera tested against TS, ES, TU, and EU extracts. (**B**) Western blot profiles of two ELISA-positive/Western blot-negative sera tested against TS, ES, TU, and EU extracts. (**C**) ELISA reactivity, expressed as an OD490-to-cutoff ratio, for discordant sera challenged with the four antigenic preparations, including negative and positive control sera. Statistical comparison among TS, ES, TU, and EU was performed using the Friedman test followed by Dunn’s multiple-comparisons test because the same initially discordant sera were evaluated under all four conditions. Positive and negative control sera are shown as contextual references and were not included in the inferential analysis. The dotted line indicates the cutoff value. TS, sonicated metacyclic trypomastigotes; ES, sonicated epimastigotes; TU, urea-extracted metacyclic trypomastigotes; EU, urea-extracted epimastigotes. ****, *p* < 0.0001.

Representative immunoblots showed broader and more intense antigen recognition with the sonicated metacyclic trypomastigote extract than with the other preparations, and ELISA performed with this extract showed clearer separation of discordant samples relative to the cut-off (Figure 3A–C). ELISA reactivity was analyzed using the Friedman test followed by Dunn’s multiple-comparisons test. A significant overall difference was observed among the four preparations (Friedman χ^2^(3) = 53.69, *p* < 0.0001). TS showed significantly greater reactivity than ES, TU, and EU (adjusted *p* < 0.0001 for all three comparisons), whereas no significant differences were observed among ES, TU, and EU. When Western blot and ELISA results were jointly considered, the sonicated metacyclic trypomastigote extract generated concordant results in 31 of 35 initially discordant sera (88.6%), compared with 19 of 35 samples (54.2%) for sonicated epimastigotes. Urea-extracted trypomastigotes and epimastigotes generated concordant results in 20 of 35 (57.1%) and 17 of 35 (48.5%) initially discordant sera, respectively (Table 1). Thus, among the antigenic preparations tested, the sonicated metacyclic trypomastigote extract produced the greatest reduction in ELISA/Western blot discordance under the experimental conditions evaluated.

### 3.4. Exploratory Low-Molecular-Weight Recognition Patterns Differed Between Cardiopathic and Non-Cardiopathic Serum Groups

To determine whether clinical presentation influenced antigen recognition, sera from patients with Chagasic cardiomyopathy were compared with sera from seropositive non-cardiopathic individuals using the four antigenic preparations evaluated in this study (TS, ES, TU, and EU). In Mexico, chronic Chagas cardiomyopathy is most commonly associated with conduction abnormalities, particularly right bundle branch block and related electrocardiographic disturbances, together with arrhythmic and structural cardiac abnormalities [32,33,34]. Consistent with this pattern, the cardiopathic serum panel used in the present study comprised 38 sera and was composed predominantly of patients with conduction system abnormalities, including incomplete right bundle branch block (BIRDHH, n = 17) and complete right bundle branch block (BCRDHH, n = 9), as well as one case of BCRDHH associated with left anterior hemiblock, three cases with left ventricular enlargement, two with left-sided enlargement, three with AQRS alterations, one with bradycardia, one with sinus bradycardia, and one with sinus rhythm; cardiomegaly was additionally documented in a subset of these patients. Individual-level demographic metadata were available only for clinically characterized cardiopathic sera and were therefore not used for comparative demographic analyses across all study groups.

**Figure 4:**
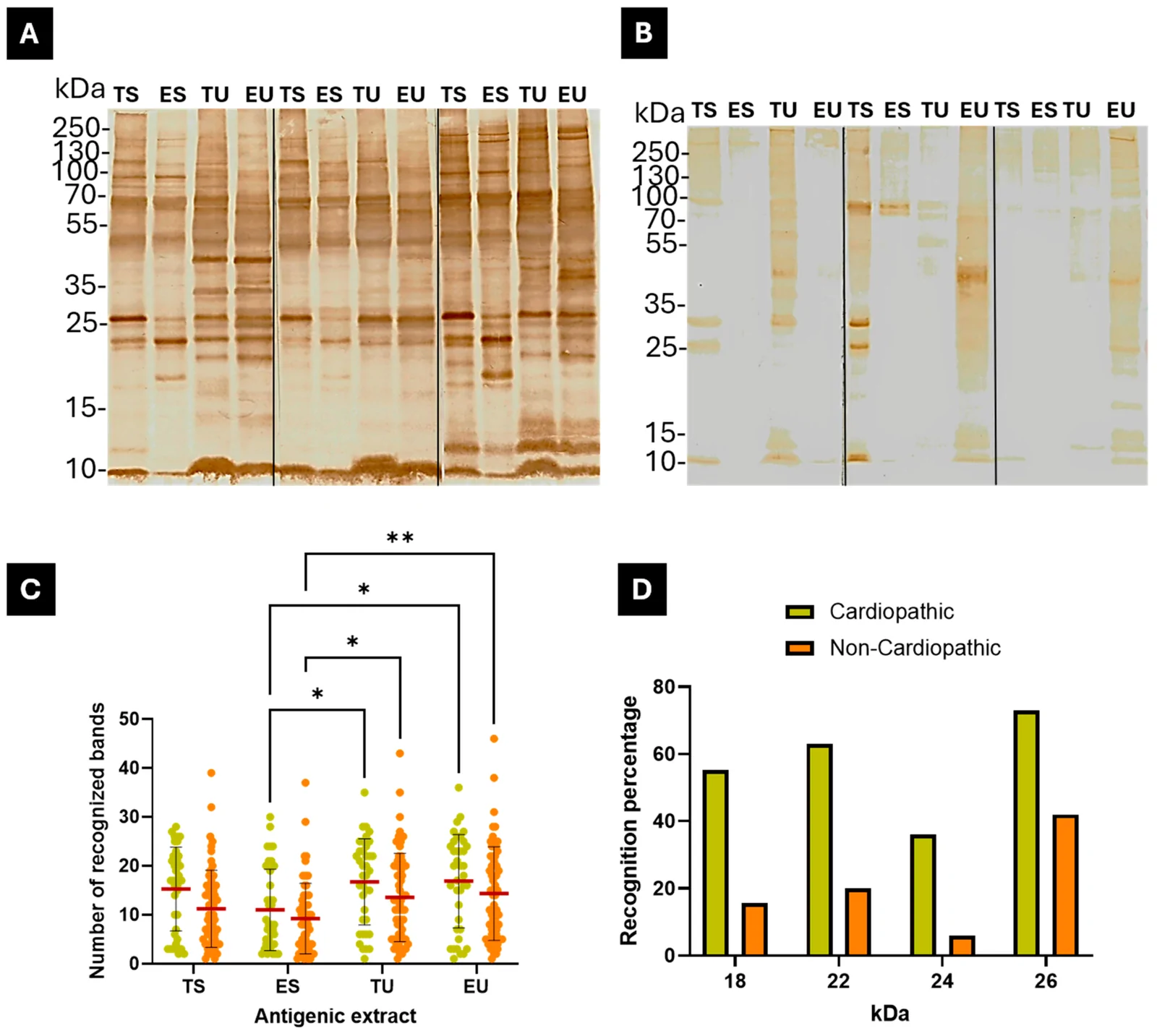
**Differential antigen recognition in cardiopathic and non-cardiopathic Chagas sera.** (**A**) Representative Western blots of sera from three patients with Chagasic cardiomyopathy tested against TS, ES, TU, and EU extracts. (**B**) Representative Western blots of sera from three seropositive non-cardiopathic individuals tested against the same extracts. (**C**) Individual distribution of the number of recognized bands according to clinical group and antigenic extract. Each dot represents one individual serum sample. Green dots indicate sera from patients with Chagasic cardiomyopathy, whereas orange dots indicate sera from seropositive non-cardiopathic individuals. Horizontal red lines and error bars indicate the mean ± SD. (**D**) Recognition frequencies of the 18, 22, 24, and 26 kDa bands. Green bars represent cardiopathic sera, and orange bars represent non-cardiopathic seropositive sera. Cardiopathic and non-cardiopathic sera were analyzed across separate gels and blots. The comparisons shown are qualitative and were not used for densitometric comparisons of band intensity across gels. TS, sonicated metacyclic trypomastigotes; ES, sonicated epimastigotes; TU, urea-extracted metacyclic trypomastigotes; EU, urea-extracted epimastigotes. * *p* < 0.05; ** *p* < 0.01.

Across the positive serum panel, antigen recognition was heterogeneous, with the greatest variability observed in the low-molecular-weight region between 45 and 10 kDa. All four extracts were immunoreactive in both clinical groups, and representative Western blots suggested broader recognition patterns in some cardiopathic sera, particularly with TS and, to a lesser extent, TU and EU (Figure 4A,B). However, group-level comparisons of the total number of recognized bands showed broad overlap and substantial dispersion between cardiopathic and non-cardiopathic sera across all four extracts (Figure 4C), indicating that chronic cardiac involvement was not associated with a uniformly higher overall antigenic burden. In line with the original dataset, 78.4% of *T. cruzi*-positive sera recognized more bands in TS than in ES, whereas the low-molecular-weight region remained the most variable portion of the immunoblot profile.

Within the TS preparation, bands with apparent molecular weights of approximately 18, 22, 24, and 26 kDa were more frequently recognized by sera from the cardiopathic group than by sera from the non-cardiopathic seropositive group (Figure 4D). Specifically, 60% of cardiopathic patients recognized three or four of these bands, compared with 22.8% of non-cardiopathic seropositive individuals. Among patients with right bundle branch block abnormalities, 72% (18/25) recognized three or four of these bands. However, these comparisons were not adjusted for age, sex, duration of infection, previous etiological treatment, or other potential confounders because complete individual-level metadata were unavailable. Therefore, the findings represent exploratory differences in band-recognition frequency within the evaluated serum groups and should not be interpreted as evidence that these bands are independently associated with Chagasic cardiomyopathy. In addition, the bands were defined solely by their apparent migration on SDS-PAGE/Western blot, and no molecular identities were assigned.

### 3.5. Sonicated Metacyclic Trypomastigote Extracts Showed the Lowest Observed Nonspecific Reactivity Among Previously Classified Seronegative Sera

**Figure 5:**
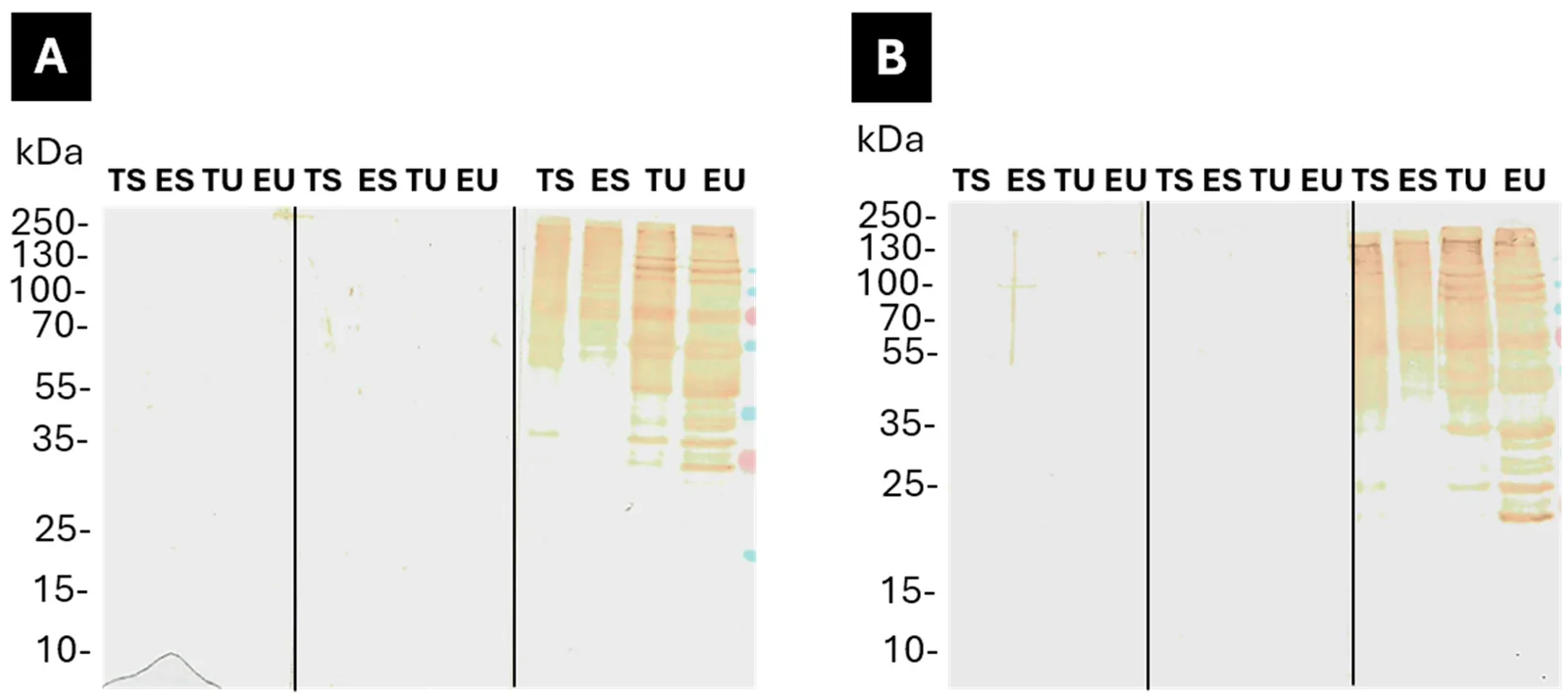
**Representative Western blot evaluation of specificity in seronegative control sera.** (**A**) Immunoblot profiles of two seronegative control sera and one *T. cruzi*-positive control serum tested against TS, ES, TU, and EU using 3.5 µg of antigen. (**B**) Immunoblot profiles of the same sera tested under increased antigen-loading conditions (10 µg of antigen). The sonicated metacyclic trypomastigote extract retained lower nonspecific reactivity than the remaining preparations. TS, sonicated metacyclic trypomastigotes; ES, sonicated epimastigotes; TU, urea-extracted metacyclic trypomastigotes; EU, urea-extracted epimastigotes.

Nonspecific reactivity was comparatively examined using previously classified seronegative control sera. Using 3.5 µg of antigen per strip, reactivity by Western blot was observed in 1/15 sera with TS (6.6%), 1/15 with ES (6.6%), 2/15 with TU (13.3%), and 4/15 with EU (26.7%) (Figure 5A; Table 2). By ELISA, the corresponding reactivity rates were 0%, 26.6%, 46.6%, and 66.6%, respectively (Table 2). Because the negative-control panel comprised only 15 sera, each reactive result substantially affected the estimated rate; therefore, these differences should be interpreted descriptively.

Using 10 µg of antigen per strip, TS again showed the lowest observed reactivity among the four preparations, with 1/15 reactive sera (6.6%), compared with 2/15 for ES (13.3%), 2/15 for TU (13.3%), and 5/15 for EU (33.3%) (Figure 5B; Table 2). These findings describe nonspecific reactivity within the evaluated serum panel and should not be interpreted as definitive estimates of diagnostic specificity. However, the small sample size and broad confidence interval for NPA preclude firm conclusions regarding superiority over the other preparations.

### 3.6. Exploratory Cross-Reactivity Analysis with a Limited Panel of Other Parasitic Infections

Because cross-reactivity is a major limitation in Chagas serology, the sonicated metacyclic trypomastigote extract was challenged with sera from patients with other parasitic infections, including toxoplasmosis (n = 3), taeniasis/cysticercosis (n = 4), and leishmaniasis (n = 15). These heterologous parasitoses were clinically and/or routinely confirmed in the laboratory at the Hospital de Infectología, Centro Médico Nacional “La Raza”, following the same serum-bank framework previously used for the in-house diagnostic standardization of Chagas serology [19].

This issue is particularly relevant for *Leishmania* spp., since *Leishmania* and *Trypanosoma cruzi* are phylogenetically related trypanosomatids that share antigenic determinants, leading to serological cross-reactivity in conventional assays. Such cross-recognition has been repeatedly described as a major obstacle to improving the specificity of Chagas serodiagnosis, especially when parasite lysates or broadly reactive native antigens are used [16,19,35,36].

**Figure 6:**
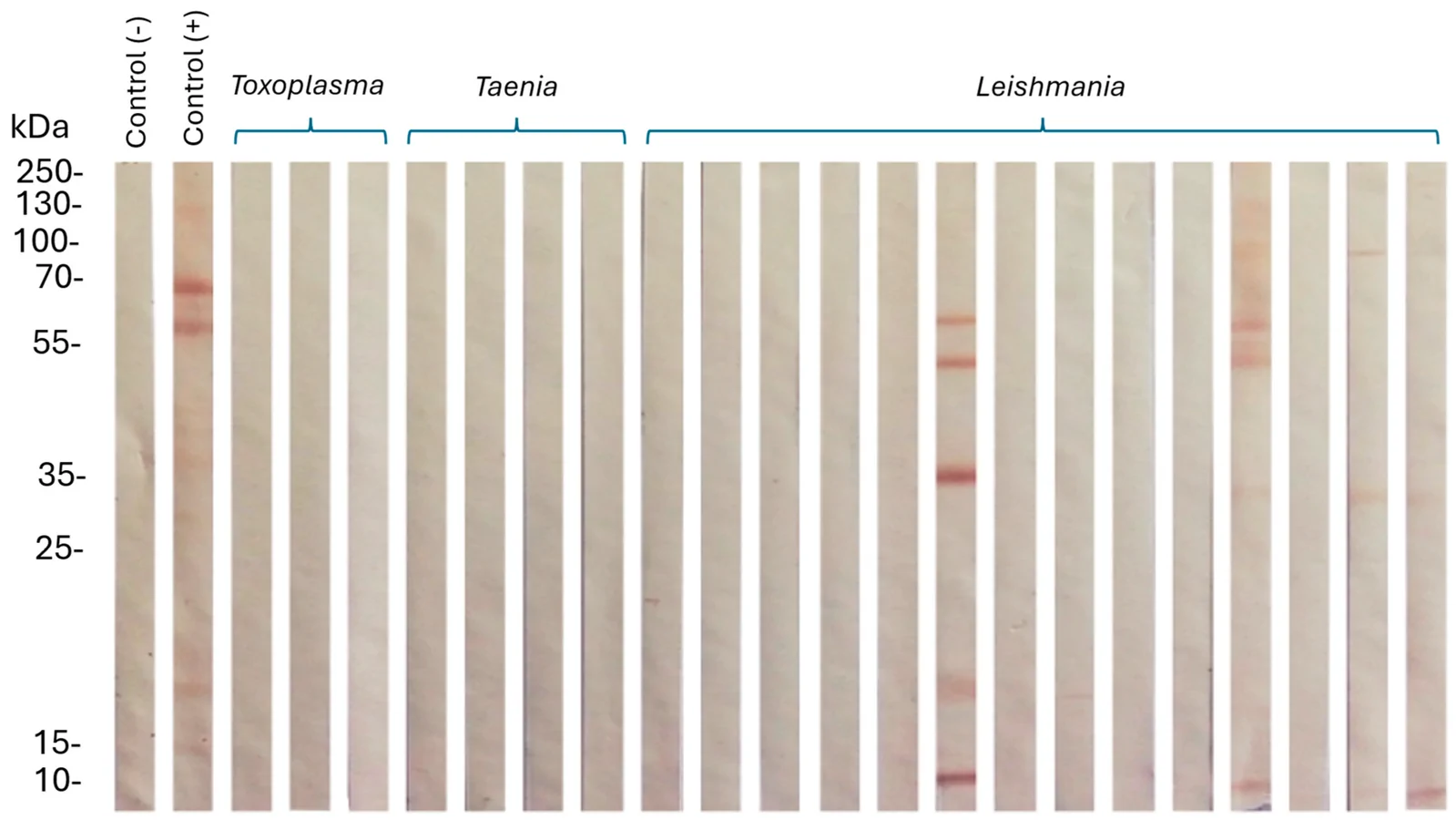
**Representative Western blot analysis of cross-reactivity with heterologous parasitic infections.** Western blot strips prepared with the sonicated metacyclic trypomastigote extract (TS) were incubated with sera from a seronegative control, a *T. cruzi*-seropositive control, and sera from patients with toxoplasmosis, taeniasis/cysticercosis, and leishmaniasis. No immunoreactivity was observed in the toxoplasmosis or taeniasis/cysticercosis sera, whereas a subset of leishmaniasis sera recognized a limited number of bands, consistent with partial cross-reactivity. Molecular weight markers (kDa) are shown on the left.

Within this limited exploratory panel, no immunoreactivity was observed with sera from patients with toxoplasmosis or taeniasis/cysticercosis (Figure 6). However, because these groups included only a small number of samples, these results should not be interpreted as definitive evidence of the absence of cross-reactivity. In contrast, a subset of sera from patients with leishmaniasis recognized a limited number of metacyclic trypomastigote-derived bands. Leishmaniasis sera recognized nine bands overall (170, 100, 70, 65, 55, 35, 20, 14, and 12 kDa), but only five of these bands (100, 70, 65, 55, and 35 kDa) were shared with sera from *T. cruzi*-positive patients (Table 3). These findings demonstrate partial heterologous recognition rather than complete elimination of cross-reactivity.

By contrast, several bands preferentially detected in Chagas sera, including those at 26, 24, 22, 18, and 10 kDa, were not recognized by *Leishmania* sera (Table 3). These findings are consistent with previous reports showing that although *Leishmania* infection can generate substantial cross-reactivity in Chagas serology, differential antigenic patterns can still be identified and exploited to improve discrimination between the two infections [35].

Thus, partial cross-reactivity with sera from patients with leishmaniasis was observed in the sonicated metacyclic trypomastigote preparation, whereas no reactivity was detected in the very small toxoplasmosis and taeniasis/cysticercosis groups. Within the evaluated panel, the recognition profiles of *T. cruzi*-seropositive and leishmaniasis sera differed in the distribution of several bands.

After adjustment for the 14 band-level comparisons using the Benjamini–Hochberg procedure, differences remained statistically significant for the 10, 18, 22, and 26 kDa bands (*q* < 0.05). The initially observed differences for the 24 and 70 kDa bands did not remain statistically significant after multiplicity correction. These results should therefore be interpreted as exploratory differential recognition frequencies within the evaluated serum panel.

Thus, although partial cross-reactivity with leishmaniasis sera remained detectable, the sonicated metacyclic trypomastigote extract retained a differential band-recognition pattern between previously classified *T. cruzi*-seropositive and leishmaniasis sera. These exploratory findings require validation in larger and more diverse heterologous serum panels.

### 3.7. Comparative Agreement Favored the Sonicated Metacyclic Trypomastigote Extract

Comparative agreement with the previous serological classification was estimated exclusively from 2 × 2 contingency analyses based on Western blot results obtained with 10 µg of antigen, using 106 previously classified *T. cruzi*-seropositive sera, including 68 seropositive individuals without documented cardiomyopathy and 38 patients with Chagasic cardiomyopathy, as well as 15 previously classified seronegative controls (Table 4). Initially discordant sera were not included in this contingency-based analysis because they constituted the unresolved group used to evaluate changes in ELISA/Western blot concordance.

Under these conditions, all four antigenic preparations showed 100% positive percent agreement, as all 106 previously classified seropositive sera were reactive by Western blot. However, negative percent agreement differed among antigenic preparations. The sonicated metacyclic trypomastigote extract showed the highest negative percent agreement, with 14 of 15 previously classified seronegative sera remaining non-reactive (93.3%). Sonicated epimastigotes and urea-extracted metacyclic trypomastigotes each showed 86.7% negative percent agreement, whereas urea-extracted epimastigotes showed 66.7% negative percent agreement (Table 4). The 95% confidence interval for the NPA of the sonicated metacyclic trypomastigote preparation was broad (68.1–99.8%), reflecting the limited number of previously classified seronegative sera. Consequently, the higher observed NPA of this preparation should be regarded as a descriptive comparative finding requiring confirmation in a substantially larger negative-control panel.

These data indicate that the comparative advantage of the sonicated metacyclic trypomastigote extract within the evaluated serum panel was not driven by increased positive agreement, which remained uniformly high across all preparations, but by its higher negative agreement and lower nonspecific recognition among previously classified seronegative sera. Because the serum categories were established using a previously standardized serological platform rather than an additional independent reference standard, these results represent reference-relative agreement and not validation of the clinical diagnostic accuracy of the new antigenic preparations. In parallel, ELISA was not included in the contingency-based agreement analysis but was used together with Western blot to evaluate changes in concordance among initially discordant sera.

## 4. Discussion

The present study shows that the comparative value of metacyclic trypomastigote-derived antigens lies not in increased positive agreement, but in lower nonspecific reactivity and greater ELISA/Western blot concordance among initially discordant sera under the conditions tested. This distinction is important because chronic *Trypanosoma cruzi* infection is still diagnosed primarily by serology, and current recommendations rely on concordant reactivity across at least 2 assays based on different principles. Therefore, discordant serology is not merely a technical inconvenience but a true diagnostic bottleneck with implications for blood bank screening, epidemiological surveillance, and treatment eligibility [30,37]. In this context, the sonicated metacyclic trypomastigote extract generated concordant ELISA/Western blot results in 31/35 initially discordant sera and showed more favorable comparative behavior than the remaining preparations (Figure 3; Table 1), supporting further evaluation of this preparation as a complementary antigen source within unresolved serological workflows. However, these findings do not establish the true infection status of the initially discordant individuals or constitute independent clinical validation of a new diagnostic assay.

A major conceptual strength of this study is that it directly addresses a longstanding inconsistency in Chagas serology: most conventional assays for human diagnosis rely on epimastigote-derived antigens, even though epimastigotes are the replicative stage in the vector and are not the form predominantly encountered by the human immune system during infection. By contrast, trypomastigotes are the bloodstream infective forms and therefore represent a biologically more relevant source of antigens for host antibody recognition [38]. This concept is indirectly supported by the high diagnostic performance of trypomastigote excreted/secreted antigens (TESA), which have repeatedly demonstrated excellent value in confirmatory immunoblot-based assays [39,40]. Our results extend that principle by showing that a whole-cell metacyclic trypomastigote sonicate can also improve the evaluation of chronic infection, particularly in the diagnostic gray zone represented by discordant sera. In that sense, the better behavior of TS relative to ES suggests that at least part of the diagnostic noise in conventional serology may derive from an antigen-source mismatch rather than from assay format.

This interpretation is reinforced by the observation that the main comparative difference associated with TS was found among previously classified seronegative sera rather than among seropositive samples. In the evaluated panel, all four preparations showed 100% PPA, whereas the sonicated metacyclic trypomastigote extract showed the highest observed NPA and the lowest numerical frequency of nonspecific recognition among the 15 previously classified seronegative sera (Table 2 and Table 4; Figure 5). However, the limited size of the negative-control panel resulted in broad confidence intervals, and each reactive result substantially affected the NPA estimates. Therefore, the numerical advantage observed for TS should be interpreted cautiously as a preliminary comparative finding rather than as conclusive evidence of greater diagnostic specificity or superiority. Larger independent negative-control panels will be required to determine the precision and reproducibility of this difference. This interpretation is also consistent with previous work from the Espinoza-Martínez group, which standardized ELISA and Western blot using Mexican parasite strains and demonstrated the importance of antigen selection for optimizing local serodiagnostic performance [19]. Likewise, later work from the same group showed that specific antigen fractions, particularly high-molecular-weight proteins, can reduce serological cross-reactivity with *Leishmania* spp. [16].

The initial serum classification was based on an in-house ELISA and Western blot platform previously standardized using 246 serum samples and Mexican *T. cruzi* strains. This platform showed high concordance with two previously standardized assays performed at the Instituto Nacional de Cardiología Ignacio Chávez (κ = 0.96) [19]. Thus, the initial classifications were not generated *de novo* for the present investigation but were derived from an established serological framework. Nevertheless, no additional independent clinical, commercial, molecular, or parasitological reference standard was applied contemporaneously to the present serum panel. Because the same general serological framework contributed to both the initial categorization and the comparative assessment of the alternative antigenic preparations, incorporation bias cannot be excluded. Accordingly, the reported PPA and NPA values should be interpreted as reference-relative agreement measures rather than as definitive estimates of diagnostic accuracy. The principal contribution of the present study is therefore comparative: under identical experimental conditions and serum-classification criteria, the sonicated metacyclic trypomastigote preparation showed lower nonspecific recognition and greater assay concordance than the other antigenic preparations evaluated.

Similarly, initially discordant sera that become concordant after retesting cannot be considered diagnostically confirmed solely by agreement between ELISA and Western blot. A change from discordant to concordant results indicates improved agreement between the assays but does not determine whether the resulting classification reflects the individual’s true infection status. Independent clinical or laboratory verification will therefore be required to establish the diagnostic meaning of these reclassifications.

The comparison between sonication and urea/thiourea/CHAPS extraction also deserves emphasis, as it illustrates that greater protein recovery does not necessarily translate into better diagnostic performance. In proteomic workflows, chaotropic agents such as urea, thiourea, and CHAPS are commonly used to enhance the solubilization of hydrophobic and membrane-associated proteins [26]. In our study, urea-based extraction indeed revealed additional bands; however, neither TU nor EU improved diagnostic performance relative to TS, and EU showed the poorest specificity. By contrast, sonication generated antigenic preparations with lower overall complexity but better confirmatory performance, particularly under TS conditions (Figure 2, Table 4). These findings suggest that, for serological applications, recovering more proteins is not equivalent to recovering more diagnostically informative proteins. Instead, the relevant issue is the balance between antigenic breadth and preserving a recognition pattern enriched in host-relevant determinants while minimizing nonspecific reactivity.

Consideration of the Querétaro strain is essential when interpreting these data. This Mexican *T. cruzi* TcI strain was selected to ensure continuity with the in-house ELISA and Western blot platform previously established using Mexican parasite antigens [18]. Because both epimastigote and metacyclic trypomastigote preparations were derived from the same strain, parasite genetic background was held constant in the direct within-study comparison. Nevertheless, only one TcI strain was evaluated, and antigenic diversity among strains and DTUs may affect antibody recognition. Therefore, the observed stage-associated differences cannot be generalized to other strains, DTUs, geographic populations, or diagnostic platforms without independent validation. However, the magnitude of this effect varied by extraction procedure: the advantage of the metacyclic preparation was more evident after sonication than after urea-based extraction, suggesting an interaction between parasite stage and extraction method rather than a universal advantage of all metacyclic trypomastigote preparations. Because only one TcI strain was evaluated, the present study cannot establish whether this stage-associated pattern is reproducible across other *T. cruzi* strains or DTUs. Comparative studies using multiple strains from TcI and other DTUs will therefore be required to determine its generalizability.

One of the strongest aspects of this study is that cross-reactivity with *Leishmania* spp., a classical and biologically expected limitation of Chagas serology, was not ignored but rather dissected in terms of banding patterns. Cross-reactivity between *T. cruzi* and *Leishmania* has been extensively documented, particularly when crude parasite lysates or native antigenic mixtures are used, because both parasites belong to the family Trypanosomatidae and share antigenic determinants [16,26,35,41]. In the previously standardized epimastigote-based platform, approximately 16% ELISA cross-reactivity with sera from individuals with leishmaniasis was reported [18]. In the present study, the sonicated metacyclic trypomastigote preparation also showed partial heterologous recognition by Western blot, indicating that using this parasite stage did not abolish cross-reactivity.

However, leishmaniasis sera recognized a restricted subset of bands, whereas several low-molecular-weight bands observed in *T. cruzi*-seropositive sera were not detected in the evaluated leishmaniasis panel (Figure 6; Table 3). Because the previous epimastigote study and the present investigation used different serum panels and analytical comparisons, these observations cannot be interpreted as a direct demonstration that TS has lower cross-reactivity than the epimastigote preparation. Rather, the current findings identify an exploratory differential recognition pattern that warrants head-to-head evaluation using the same heterologous serum panel.

No reactivity was observed with the three toxoplasmosis and four taeniasis/cysticercosis sera included in the present study. However, these sample sizes are insufficient to establish the absence of cross-reactivity, and other potentially interfering infections were not evaluated. Therefore, the present evidence must be restricted to the specific samples and infections tested and should not be extrapolated to a broader heterologous population.

The cardiopathy-related findings should be interpreted as exploratory. At the group level, cardiopathic and non-cardiopathic sera showed substantial overlap in the total number of recognized bands, indicating that cardiac involvement was not associated with a uniformly broader antigen-recognition profile (Figure 4C,D). Nevertheless, within the sonicated metacyclic trypomastigote preparation, bands with apparent molecular weights of approximately 18, 22, 24, and 26 kDa were more frequently recognized by sera from the cardiopathic group, particularly among individuals with right bundle branch block abnormalities. However, complete individual-level information on age, sex, infection duration, previous etiological treatment, and other potentially relevant clinical variables was unavailable across the study groups. Consequently, multivariable adjustment could not be performed, and the observed recognition pattern cannot be independently attributed to the cardiac phenotype. These findings should therefore be regarded as an unadjusted, hypothesis-generating observation rather than evidence of validated cardiomyopathy-associated biomarkers.

The molecular identities of the 18, 22, 24, and 26 kDa bands cannot be established based on their apparent migration on SDS-PAGE and Western blot, as multiple proteins may migrate within similar molecular-weight ranges. Nevertheless, antigens with overlapping apparent molecular weights have been described previously in *T. cruzi*. Tc24 is a 24 kDa flagellar calcium-binding protein that has been evaluated as a recombinant antigen and vaccine candidate [42,43]. Previous studies using Mexican parasite isolates have also reported immunodominant components within a similar molecular-weight range [44], and sera from naturally infected dogs showed recognition of an approximately 26 kDa antigen along with other major antigenic bands [45]. These reports provide a rationale for prioritizing the corresponding gel regions for future molecular characterization, but they do not demonstrate that the bands observed in the present study correspond to Tc24 or to any previously described antigen. Their identities therefore remain undetermined and will require proteomic analysis followed by recombinant or antigen-specific validation. The identities of the approximately 18 and 22 kDa bands are likewise unknown.

A broader implication of this study is that it connects native-stage antigen discovery with the current shift toward recombinant and chimeric diagnostic platforms. The most promising contemporary assays for chronic Chagas disease increasingly rely on recombinant multiepitope or chimeric antigens because they offer superior standardization, scalability, and specificity [46,47]. Bertha Espinoza has also contributed to this direction through studies evaluating chimeric antigens for Chagas diagnosis in non-endemic settings [48]. Rather than competing with those technologies, the present findings help explain why some antigenic systems perform better than others: the improved confirmatory behavior of TS indicates that biologically relevant trypomastigote determinants remain underrepresented in many conventional epimastigote-based preparations. In this sense, the TS extract can be viewed not only as a potential confirmatory reagent but also as a discovery platform for identifying the subset of native trypomastigote antigens to be prioritized for future recombinant or chimeric assay development.

This study has several strengths. It compares two parasite stages and two extraction strategies in parallel; it examines not only positive and negative sera but also discordant clinical samples, which are often excluded from diagnostic studies; it evaluates cross-reactivity with relevant heterologous parasitoses; and it links serological recognition with clinically stratified phenotypes. At the same time, several limitations should be acknowledged. First, the serum panel originated from two collaborating clinical institutions in central Mexico (Centro Médico Nacional “La Raza” and the Instituto Nacional de Cardiología Ignacio Chávez) and was subsequently centralized in the serum bank of the Instituto de Investigaciones Biomédicas, UNAM. Therefore, although the samples were not derived from a single recruitment center, the cohort remained geographically restricted to central Mexico and was evaluated within a single reference-laboratory framework. The generalizability of the findings to geographically distinct populations, different epidemiological settings, and external laboratories remains to be established. Complete demographic and treatment metadata were also unavailable for some study groups. Second, positive and negative classification was based on previous serological criteria, which is appropriate for chronic Chagas disease but may introduce verification or incorporation bias when estimating diagnostic performance. Third, only 15 previously classified seronegative sera were included. Consequently, each reactive result produced a substantial change in the NPA estimate, and the corresponding confidence intervals were broad. The lower observed nonspecific reactivity of TS therefore requires confirmation in a larger independent negative-control panel before any conclusion regarding comparative superiority can be drawn.

Fourth, the heterologous serum panel was limited, particularly for toxoplasmosis and taeniasis/cysticercosis, and did not include the broader range of infections that may interfere with Chagas serology. Although partial recognition by leishmaniasis sera and a differential band distribution were observed, the study was not designed to establish absence or general reduction in cross-reactivity. A direct head-to-head comparison of TS and conventional epimastigote preparations using the same expanded heterologous panel will be necessary. Fifth, only the Mexican Querétaro TcI strain was evaluated. Although using the same strain for epimastigote and metacyclic trypomastigote preparations controlled the parasite genetic background in within-study stage comparisons, the reproducibility of the observed stage-associated effect across other strains and DTUs could not be assessed.

Sixth, the low-molecular-weight bands enriched in cardiopathic sera were assigned only by apparent molecular weight and were not molecularly characterized. Although all sera had previously been characterized through concordant conventional ELISA and Western blot testing, this previous testing did not constitute a technical replicate of the current four-antigen comparison. Cardiopathic and non-cardiopathic sera were analyzed across separate gels and blots, and systematic technical replication of the present immunoblot analysis was not performed. Therefore, residual gel-to-gel variability cannot be excluded, and the clinical-group comparison should be considered exploratory. Finally, the study was not designed to benchmark TS directly against the best-performing recombinant, chimeric, or commercial diagnostic assays currently available [46,47]. In addition, information on concomitant illnesses was incomplete for the archived sera. Therefore, we cannot exclude that unrecorded comorbid conditions contributed to atypical or nonspecific serological reactivity in some samples. Even with these limitations, the present data provide strong proof of concept that stage-informed antigen selection can improve the confirmatory resolution of chronic Chagas serology.

The present findings provide proof-of-concept evidence that metacyclic trypomastigote sonicates may contain antigenic determinants that improve comparative assay behavior within unresolved chronic Chagas serology. Their immediate value lies in demonstrating that parasite stage and extraction method influence nonspecific reactivity and ELISA/Western blot concordance. Their longer-term discovery value lies in identifying stage-associated native targets that could subsequently be incorporated into standardized recombinant or chimeric immunoassays. Before any clinical application can be considered, these observations must be independently validated using geographically distinct cohorts, broader heterologous infection panels, additional *T. cruzi* strains and DTUs, external laboratories, and molecularly characterized antigens.

## 5. Conclusions

This study provides proof-of-concept evidence for the comparative value of metacyclic trypomastigote-derived antigens, particularly those obtained by sonication, in the evaluation of initially discordant chronic Chagas disease serology. Their main advantage was not increased positive percent agreement, which remained uniformly high across all preparations under the conditions tested, but rather the highest observed negative percent agreement, the lowest numerical frequency of nonspecific recognition within the limited seronegative panel among previously classified seronegative sera, and greater ELISA/Western blot concordance after retesting initially discordant samples. In this regard, the sonicated metacyclic trypomastigote extract showed the most favorable comparative behavior among the preparations evaluated. Because the epimastigote and metacyclic trypomastigote preparations were derived from the same Querétaro TcI strain, parasite genetic background was held constant in the direct comparison between life-cycle stages. Therefore, the observed difference supports a stage-associated effect within the Querétaro TcI background, particularly under sonication conditions. However, because only one strain was evaluated, this finding should not be extrapolated as a general property of metacyclic trypomastigotes from other strains or DTUs.

Despite partial cross-reactivity with *Leishmania* spp., this extract retained a differential immunoblot pattern within the limited heterologous panel tested. The preferential recognition of selected low-molecular-weight bands in sera from patients with Chagasic cardiomyopathy should be interpreted as an exploratory finding, as these bands were assigned only by apparent molecular weight and were not molecularly identified. The increased concordance observed after retesting does not establish the true infection status of initially discordant individuals, and the PPA and NPA estimates represent agreement with a previously standardized serological classification rather than diagnostic accuracy against an independent reference standard.

Taken together, these findings support the concept that stage-informed antigen selection influences serological assay behavior and identify sonicated metacyclic trypomastigote extracts as a candidate antigen source for further comparative validation and as a discovery platform for potentially informative stage-associated antigens, rather than as a clinically validated diagnostic reagent. Independent validation using geographically distinct cohorts, external laboratories, larger negative-control groups, broader heterologous infection panels, additional *T. cruzi* strains and DTUs, and molecular characterization of the discriminatory bands will be required before clinical implementation can be considered.

## Figures and Tables

**Table 1:** **ELISA/Western blot concordance among initially discordant sera according to antigenic preparation.**

Parameter	TS	ES	TU	EU
Total discordant sera evaluated	35	35	35	35
ELISA−/WB+ in discordant panel	27	27	27	27
ELISA+/WB− in discordant panel	8	8	8	8
Initially discordant sera achieving concordant results, n/N	31/35	19/35	20/35	17/35
Post-retesting concordance rate (%)	88.6	54.2	57.1	48.5
Remaining discordant in the ELISA−/WB+ subgroup	3	13	14	14
Remaining discordant in the ELISA+/WB− subgroup	1	3	1	4

Note: Concordant results were defined as agreement between ELISA and Western blot after retesting with the same antigenic preparation, either as concordant reactive or concordant non-reactive results, according to the predefined ELISA cutoff and Western blot interpretation criteria. Sera remaining discordant were those that continued to show non-concordant ELISA and Western blot results after retesting.

**Table 2:** **Reactive results obtained with the antigenic preparations among previously classified seronegative control sera.**

Antigenic Extract	Western Blot, 3.5 µg	ELISA	Western Blot, 10 µg
Sonicated metacyclic trypomastigotes (TS)	1/15 (6.6%)	0/15 (0%)	1/15 (6.6%)
Sonicated epimastigotes (ES)	1/15 (6.6%)	4/15 (26.6%)	2/15 (13.3%)
Urea-extracted metacyclic trypomastigotes (TU)	2/15 (13.3%)	7/15 (46.6%)	2/15 (13.3%)
Urea-extracted epimastigotes (EU)	4/15 (26.7%)	10/15 (66.6%)	5/15 (33.3%)

Note: Values are shown as the number of reactive results among 15 previously classified seronegative control sera, followed by the corresponding percentage. TS, sonicated metacyclic trypomastigotes; ES, sonicated epimastigotes; TU, urea-extracted metacyclic trypomastigotes; EU, urea-extracted epimastigotes. Reactivity rates were calculated among sera classified as seronegative using the previously standardized in-house serological framework and should not be interpreted as false-positive rates established against an independent reference standard. The sonicated trypomastigote extract consistently showed the lowest nonspecific reactivity across assay formats.

**Table 3:** **Differential antigen recognition by sera from patients with** ***T. cruzi*** **and** ***Leishmania*** **infections against the sonicated metacyclic trypomastigote extract.**

Band (kDa)	*T. cruzi* Sera (n = 20), % Recognition	Leishmaniasis Sera (n = 15), % Recognition	Observed Pattern	Fisher’s Exact *p*-Value	BH-Adjusted *q*-Value
170	0	6.7	*Leishmania*-associated	0.429	0.429
100	30	13.3	Shared	0.419	0.429
70	40	6.7	Shared	0.048	0.113
65	35	13.3	Shared	0.244	0.342
55	40	13.3	Shared	0.134	0.268
35	40	20.0	Shared	0.281	0.358
20	0	13.3	*Leishmania*-associated	0.176	0.275
14	0	13.3	*Leishmania*-associated	0.176	0.275
12	0	6.7	*Leishmania*-associated	0.429	0.429
26	45	0	Chagas-associated	0.004	**0.020**
24	30	0	Chagas-associated	0.027	0.075
22	40	0	Chagas-associated	0.006	**0.020**
18	40	0	Chagas-associated	0.006	**0.020**
10	75	0	Chagas-associated	<0.001	**<0.001**

Note: Values represent the percentage of sera recognizing each band in Western blot assays performed with 3.5 µg of sonicated metacyclic trypomastigote extract. Two-sided Fisher’s exact tests were used to compare recognition frequencies between previously classified *T. cruzi*-seropositive sera and sera from patients with leishmaniasis. Because 14 bands were evaluated, raw *p*-values were adjusted using the Benjamini–Hochberg false-discovery rate procedure. Both raw *p*-values and adjusted *q*-values are presented, and *q* < 0.05 was considered statistically significant. The classifications in the interpretation column refer exclusively to the recognition patterns observed within the evaluated serum panel. Bold values indicate bands showing statistically significant differences in recognition between the two parasitoses after Benjamini–Hochberg adjustment (*q* < 0.05).

**Table 4:** **Comparative agreement of the four antigenic preparations with the previous serological classification based on Western blot using 10 µg of antigen.**

Antigenic Extract	TP	FN	FP	TN	PPA % (95% CI)	NPA % (95% CI)
TS	106	0	1	14	100.0 (96.6–100.0)	93.3 (68.1–99.8)
ES	106	0	2	13	100.0 (96.6–100.0)	86.7 (59.5–98.3)
TU	106	0	2	13	100.0 (96.6–100.0)	86.7 (59.5–98.3)
EU	106	0	5	10	100.0 (96.6–100.0)	66.7 (38.4–88.2)

Note: Agreement matrices were constructed from Western blot results obtained with 10 µg of antigen using 106 sera previously classified as seropositive and 15 sera previously classified as seronegative by the standardized in-house serological platform. Exact 95% confidence intervals (CIs) were calculated using binomial methods. PPA and NPA describe agreement with the previous serological classification and should not be interpreted as diagnostic sensitivity or specificity against an independent reference standard. Initially discordant sera were not included because they constituted the group used to assess changes in ELISA/Western blot concordance. Sera from other parasitic infections were not included because they were evaluated only with the sonicated metacyclic trypomastigote preparation. TS, sonicated metacyclic trypomastigotes; ES, sonicated epimastigotes; TU, urea-extracted metacyclic trypomastigotes; EU, urea-extracted epimastigotes; PPA, positive percent agreement; NPA, negative percent agreement.

## Data Availability

The original contributions presented in this study are included in the article/Supplementary Material. Further inquiries can be directed to the corresponding authors.

## Supplementary Materials

The following supporting information can be downloaded at: https://www.mdpi.com/article/10.3390/microorganisms14091899/s1. The available original, uncropped Western blot scans recovered from the archived dataset, including those corresponding to the representative panels shown in Figure 3, Figure 4, Figure 5 and Figure 6.
